# Commentary: Physical time within human time

**DOI:** 10.3389/fpsyg.2023.1096890

**Published:** 2023-07-13

**Authors:** Dennis Dieks

**Affiliations:** History and Philosophy of Science, Descartes Centre, Utrecht University, Utrecht, Netherlands

**Keywords:** physical time, human time, perspectivalism, information, quantum mechanics

## 1. Introducton: physical vs. human time

Buonomano and Rovelli (2021) and Gruber et al. (2022) emphasize that time as it figures in physics is different from time as we experience it. Physics provides us with an analysis of temporal features of the world that are independent of whether or not there are observers, whereas experiential time is private and subjective. Moreover, experiential time possesses properties that seem completely absent from physical time. For example, our temporal experience is dynamic, characterized by a privileged instant on the time axis, the Now, that continuously shifts from Past to Future. Physics does not recognize such a privileged moment, and motion of time itself (as opposed to ordinary motion, i.e. change of spatial position as a function of time) seems even impossible to define from a physical point of view.

Buonomano and Rovelli (2021) and Gruber et al. (2022) argue that these differences are not in conflict with the universality claim of physics: physical time may without contradictions be assumed to govern us and other organisms no less than elementary particles, planets and stars. However, the way we (and other organisms) experience time is not only determined by the nature of physical time, but also by how we process it and how we represent it to ourselves. The nature of human time consequently at least partially depends on the organization of our sensory system and brain.

To understand phenomenological time we therefore have to invoke neuroscience and psychology. We must consider how organisms process information coming in from events in the external world and how organisms internally represent that information. For this purpose Hartle (2005) first introduced a simple model of an “Information Gathering and Utilizing System” (IGUS). Hartle's IGUS contains a number of registers, one for novel information and some for storing data about the past. In the IGUS the contents of these registers are constantly updated and compared—see, e.g., Callender (2017, chapter 11). Gruber et al. (2022) present an overview of the architecture of more sophisticated IGUSs, with details about how they could explain features of human time, in particular our experience that time *flows*.

## 2. The Now and perspectives

A key factor in the explanation of our time experience is that our Now is not point-like but has a finite duration (the “specious present”). This implies that information from different temporal stages of an observed process can be part of the same experienced moment, which makes it possible to be “instantaneously” aware of change. This is relevant for the explanation of our awareness of time flow: the presence of differences between successive stages of a process during one specious moment may be responsible for a “state of tension” associated with a subjective feeling of flow.

Another point to be explained is our intuition that our Now is spatially extended, so that it makes sense to speak about the global state of the world around us *now*, at any given moment. Perhaps surprisingly, modern physics denies the objectivity of such a global now and the global simultaneity on which it relies. Nevertheless, physics is capable of explaining our intuition: Things around us typically change little during the time needed by light signals coming from them to reach us and this creates the impression of instantaneous contact even with objects at a distance. However, in reality physical information transfer takes time, so that we actually are in contact with the past. That our experiential global Now in reality corresponds to a physical time window during which we receive information from the past is not difficult to understand and accept, however, and it is not impossible for us to adapt our intuitions accordingly. This is a step that brings experiential time and physical time closer together.

Another essential feature of experiential time, however one less frequently discussed, is that it is *perspectival*: all our temporal judgements are made from our personal vantagepoint. This “subjective” aspect of experience, the fact that it always presupposes a “point of view,” is shared by all experiential qualia. This unavoidable perspective-dependence has frequently been used to argue that there exists an unsurmountable barrier between human experience and the objective, perspective-less facts of physics, with the consequence that experiential facts cannot be reduced to physical facts (see Nagel, 1974 for a famous argument along these lines). The validity of the argument is not uncontroversial, but in any case it is interesting to note that during the last decades the notion of judgements and descriptions that are inseparable from a vantagepoint has been gaining prominence even within fundamental physics. Partly, this is because it has become more popular, especially in quantum mechanics, to interpret theories as practical tools, used by agents, rather than as objective descriptions of nature. If a view of this kind is accepted, human perspectives automatically become important. But this is not the only way perspectives have entered physics: it has been proposed that perspectives are even essential in more traditional views, according to which physical theories are not merely tools but provide objective descriptions of the world, quite independently of the presence of observers or human agents.

## 3. Perspectives in physics

The view that physical theories are merely instruments is exemplified by QBism, a recent interpretation of quantum mechanics. According to QBism it is not the aim of quantum mechanics (or even of physics in general) to provide a true representation of the external world. Rather, the states that are assigned to physical systems, the mechanisms that are judged to apply, and the predictions that are made are all taken to represent beliefs of agents using the theory. Accordingly, all quantum descriptions and predictions become relative to human users. That human time becomes primary is one of the consequences.

A core motivation for this “subjectivist” position is the wish to create room for the possibility that different agents adopt completely different beliefs about situations in the physical world. A divergence of subjective points of view is of course nothing unusual. But in quantum mechanics there are reasons to think that there are perspectival differences that should be recognized even if one does not subscribe to QBism and its subjectivity. This motivation for perspectivalism is illustrated by situations of the “Wigner's Friend” type.

In Wigner's Friend scenarios an experimenter in a hermetically sealed laboratory (the “Friend”) successfully performs a measurement and finds one definite result. However, an external observer (“Wigner”), who cannot receive information from within the laboratory, is licensed by quantum mechanics to describe the lab and its contents with a “superposition” state, in which all possible internal measurement results are represented and in which his friend's actual result is not privileged. This superposition of all possibilities is different from what common sense would lead us to expect, namely a state of ignorance about the actual internal outcome. The quantum superposition corresponds to a situation in which there *is* no definite outcome inside. The outside observer can verify, experimentally, that his external view involving a superposition is correct; but this view does not dovetail with the internal description, which can also be verified by experiments, but this time *within* the sealed laboratory. Cases like this suggest a perspectivalism in which different agents arrive at descriptions that do not fit together but are equally valid from their own points of view.

Despite this formulation in terms of observers who perform experiments, the perspectivalism under discussion is meant to have an objective meaning. One may replace Wigner and his friend by inanimate measuring devices, or information processing systems in the sense of IGUSs. One may even go further, and think of elementary physical systems that do not possess the internal IGUS information processing capabilities. This leads to the idea that physical properties quite generally are perspectival, in the sense of being defined only as relative to other physical systems.

This proposal goes back at least to Everett's “relative state interpretation” of quantum mechanics and was later developed by others (Everett, 1957; Rovelli, 1996; Laudisa and Rovelli, 2013; Dieks, 2022). Accordingly, properties of physical systems have the logical status of relations rather than of monadic properties. It is not only that the *values* of physical quantities may vary depending on the reference system from which they are judged—that would be unremarkable, familiar as it is from classical physics and daily life. In quantum mechanics a much stronger perspectivalism manifests itself, according to which it may be perspective-dependent *which* quantities possess definite values at all. Thus, in the Wigner's Friend situation the quantity internally measured has a definite value, after the measurement, for the friend but not for Wigner who is outside the lab.

## 4. Discussion

The relevance of all this for the comparison of physical and human time is that most ingredients of human time may already be present on the most fundamental physical level. For the IGUS-Now and its dynamism it was crucial that the connections to the outside world did not remain constant over specious presents. Something very much like this occurs quite generally in the physical world, regardless of whether the systems involved are complicated enough to mimic the functioning of IGUSs, with their registers. Physical information transfer requires interactions and transformations, in a process that takes a certain time—it is not possible to have an impact on a physical system in a literally size-less point-event. Processes of change require a “physical specious present” of finite extent.

As we have indicated, quantum mechanics gives us reasons to think that the information received by a physical system has a relational character: it is specific to the system's perspective, and in this sense not shareable. It is seductive to see here at least an analogy to the private character of human qualia, and in particular to the subjective experience of time flow.

The differences between human time and physical time may therefore be even smaller than argued by Gruber, Block and Montemayor, Bonomano and Rovelli, and others. It is true, evidently, that elementary particles do not have an internal representation of time flow as humans have. For that, a more complicated architecture like that of the IGUSs with their registers seems necessary. But temporal relations with the same structure as those determining experiential time seem to be present even on the level of fundamental physical systems.

## Author contributions

The author confirms being the sole contributor of this work and has approved it for publication.
